# Development and pilot testing of a nurse-led common-sense model of self-regulation-based heart failure self-care program

**DOI:** 10.1186/s12912-025-02722-9

**Published:** 2025-01-23

**Authors:** Zehao Huang, Sek Ying Chair

**Affiliations:** The Nethersole School of Nursing, Faculty of Medicine, The Chinese University of Hong Kong, 8/F, Esther Lee Building, Horse Material Water, Shatin, New Territories, Hong Kong SAR China

**Keywords:** Heart failure, Self-care, Intervention development, Pilot study

## Abstract

**Background:**

Self-care practices among people with heart failure (HF) remain suboptimal. Nurse-led self-care interventions hold promise in managing this condition. The Common-Sense Model (CSM) of Self-Regulation is a widely adopted theoretical framework that promotes behavior change and improves disease prognosis among patients. Therefore, this study aimed to describe the development and pilot testing of a nurse-led CSM of Self-Regulation-based self-care intervention among people with HF.

**Methods:**

Intervention development was informed by a comprehensive review of the relevant literature, the CSM of self-regulation, international and national guidelines, and findings from our previous systematic reviews. The pilot study utilized a single-blinded, two-arm, parallel group, randomized controlled trial (RCT) design, adhering to the CONSORT Statement. Eligible participants were randomly assigned at a 1:1 ratio to either the intervention group or the control group. Data were collected at baseline and immediately after the intervention, with a focus on evaluating feasibility, acceptability, and potential effects.

**Results:**

We developed and validated a nurse-led, theory-driven, evidence-based, and need-oriented HF self-care program. A total of 26 participants were enrolled in the pilot study, achieving an eligibility of 79.4%, a recruitment rate of 96.3%, and a retention rate of 92.3%. Most participants (83.3%) recognized the benefits of the intervention. The intervention significantly improved illness perceptions, self-care self-efficacy, and self-care behaviors among people with HF.

**Conclusion:**

The nurse-led CSM of Self-Regulation-based self-care intervention is feasible, acceptable, and potentially beneficial for people with HF. A full-scale mixed-method RCT is recommended to further examine the intervention’s effectiveness.

**Trial registration:**

Chinese Clinical Trial Registry (No., ChiCTR2300068156; February 9, 2023).

**Supplementary Information:**

The online version contains supplementary material available at 10.1186/s12912-025-02722-9.

## Background

Heart failure (HF) is a widespread and burdensome health concern that significantly impacts affected individuals, leading to compromised health-related quality of life, increased symptom burden, sleep disturbances, psychological distress, and increased rates of hospitalization and mortality [1, 2]. A large national survey in China revealed that 1.3% of individuals aged 35 years or older (approximately 13.7 million) are living with HF [3]. Effective HF self-care has been shown to lead to a better prognosis for patients [4]; however, self-care performance remains insufficient among Chinese patients with HF [5, 6]. Therefore, it is essential to develop and implement effective strategies to promote self-care among people with HF.

Extant literature has demonstrated the promise of nurse-led self-care interventions in the management of HF [7, 8]. These interventions are led primarily by nurses but may also include input from other relevant personnel, aiming to help individuals, families, and communities promote health, prevent disease, maintain health, and cope with illness and disability [9, 10]. Despite the widespread dissemination of information on self-care, the uptake of self-care activities remains low, highlighting the need to go beyond viewing self-care as just a knowledge issue [11]. Therefore, when designing appropriate nurse-led self-care interventions, it is crucial to consider the multifaceted factors that can enhance self-care behaviors.

The values of the Common-Sense Model (CSM) of Self-Regulation in promoting behavior change and improving disease prognosis among patients has been recognized [12–18]. The CSM is a theoretical framework used to understand illness self-management [19]. This model is characterized by three main constructs: illness perceptions, coping procedures, and appraisal [20]. According to the model, individuals facing health threats develop cognitive and emotional illness perceptions that can affect their disease outcomes directly or indirectly, with the latter having coping procedures as mediators. Appraisals provide dynamic feedback, allowing for updates to illness perceptions and coping procedures on the basis of changes in disease outcomes [21]. In addition, self-efficacy plays a critical moderating role in the relationship between illness perceptions and coping behaviors [21, 22]. Therefore, when interventions are developed to improve self-care and health outcomes for those living with HF, both illness perceptions and self-efficacy is essential. Illness perceptions are the core components of CSM, representing cognitive and emotional responses to health threats that trigger reactions [21]. Self-efficacy significantly influences a person’s engagement in behaviors and the effort they exert in practicing them [23].

Prior to this study, two randomized controlled trials based on this model were conducted among people with HF [12, 18]. These studies focused solely on cognitive perceptions and neglected the emotional components. Additionally, they did not adequately incorporate effective behavior change techniques. Important outcomes such as symptom burden, sleep quality, depression, anxiety, healthcare service utilization, and mortality were also not explored. Consequently, further research is warranted to fill these notable knowledge gaps.

The United Kingdom Medical Research Council (MRC) framework is adopted as the methodological guidance for our study [24]. This framework includes four phases: developing the intervention, assessing feasibility, evaluating the intervention, and implementing it. This study focuses on the development and pilot testing of a nurse-led CSM of Self-Regulation-based HF self-care program.

## Methods

### Intervention development

The development of nurse-led CSM of Self-Regulation-based HF self-care program was informed by a review of the relevant literature, the theoretical foundations of CSM of self-regulation, international and national guidelines, and the findings of our previous systematic reviews.

### Pilot study

**Aims.** This study aimed to assess the feasibility, acceptability, and potential effects of the nurse-led CSM of Self-Regulation-based self-care intervention among people with HF.

**Study design.** This study is a single-blinded, two-arm, parallel group, randomized controlled trial (RCT). Eligible participants were randomly assigned at a 1:1 ratio to either the intervention group or the control group via block randomization generated by an independent researcher. Allocation concealment was ensured with sequentially numbered, opaque sealed envelopes. A research nurse, blinded to the group assignments, collected the post-test data. The participants in the intervention group received a 6-week nurse-led CSM of Self-Regulation-based HF self-care program (details were presented in the result section) in addition to usual care (routine treatment and nursing, health education, and a telephone follow-up in the first week after the patient is discharged from hospital), whereas those in the control group received usual care only. Data were collected at baseline and immediately post-intervention. We evaluated the program’s feasibility, acceptability, and potential effects, and the study followed the CONSORT statement [25].

**Setting and subjects.** Participants were recruited using convenience sampling method in February 2023 at a university-affiliated hospital in China. HF patients admitted to the hospital were enrolled if they met the following criteria: (1) adults (aged ≥ 18 years), (2) clinically diagnosed with HF, (3) classified as New York Heart Association (NYHA) functional class I-IV, (4) able to complete the questionnaire, (5) able to communicate in Mandarin or Cantonese, (6) available for telephone follow-up and (7) agreed to participate in the study. Subjects were excluded if they had psychiatric diseases, severe physical illness other than HF, or were concurrently involved in other clinical trials. Hertzog (2008) [26] suggested that a minimum of 10 to 15 participants per group is needed for a pilot study. Thus, the sample size for this pilot study was set at 26 participants (13 in each group).

**Outcome measures and data collection.** The feasibility outcomes included (1) subject recruitment and retention: the eligibility rate, recruitment rate, retention rate, attrition rate, and the reasons for dropping out of the study; (2) intervention implementation: the time taken to complete each session, the percentage of participants who complete all the sessions, and reasons for discontinuing the sessions; and (3) data collection: the time taken to complete measures. The acceptability of the intervention was assessed at the end of the study using a self-designed questionnaire. The questionnaire gathered data on the participants’ perceptions of the intervention content, dosage, materials, and effects. Each item of the questionnaire was rated as strongly disagree, disagree, neither agree nor disagree, agree, or strongly agree. The intervention was considered acceptable if over 80% of participants rated it as “agree” or “strongly agree” for all items. Sociodemographic and clinical information and outcome variables were also collected. Sociodemographic data collected included age, gender, marital status, educational level, employment status, place of residence, living condition, smoking status, and alcohol intake status. The required clinical information included cause of HF, NYHA functional class, time since diagnosis, left ventricular ejection fraction, comorbidity (measured by the Charlson Comorbidities Index), number of hospitalizations, body mass index, blood pressure, heart rate, NT-ProBNP, total cholesterol, triglyceride, high density lipoprotein cholesterol, low density lipoprotein cholesterol, urea, creatinine, HbA1C, and frailty (measured by the Tilburg Frailty Indicator). The study outcomes included illness perceptions (measured by the 9-item Brief Illness Perception Questionnaire [27]), self-care self-efficacy (measured by the 10-item Self Care Self-Efficacy Scale [28]), self-care behaviors (measured by the 29-item Self-Care of Heart Failure Index version 7.2 [29]), health-related quality of life (measured by the 21-item Minnesota Living with Heart Failure Questionnaire [30]), depression (measured by the 9-item Patient Health Questionnaire [31]), anxiety (measured by the 7-item Generalized Anxiety Disorder [32]), symptom burden (measured by the Memorial Symptom Assessment Scale-Heart Failure [33]), sleep quality (measured by the Pittsburgh Sleep Quality Index [34]), healthcare service utilization (the number of HF-related hospital readmissions, HF-related hospital days, HF-related emergency room visits, and HF-related unscheduled outpatient department visits), and mortality. Before the data collection, all eligible participants were informed of the purpose of the study and asked to sign the informed consent form. The principal investigator collected the baseline data through face-to-face interviews and the medical system, whereas a research nurse conducted post-test data collection via telephone interviews.

**Data analysis.** Statistical analyses were conducted using IBM SPSS version 27.0. A two-sided *p* < 0.05 was considered statistically significant. Normally distributed continuous variables are presented as the means and standard deviations, while non-normally distributed variables are described as medians and interquartile range (IQR). Categorical variables are presented as frequency and percentage. The intention-to-treat principle was used to analyze the data. The homogeneity between groups was tested by an independent t‐test or Fisher’s exact test as appropriate. Generalized estimating equation (GEE) models were used to compare the differential changes in outcome variables including illness perceptions, self-care self-efficacy, self-care behaviors, quality of life, depression, anxiety, symptom burden, and sleep quality between groups across the time points. The Mann–Whitney U test was used to compare the number of HF-related hospital readmissions between the intervention and control groups.

## Results

### Development of study intervention

**Concept of HF self-care.** The concept of HF self-care was identified and conceptualized according to the Situation-Specific Theory of Heart Failure Self-Care [35]. As described in this theory, HF self-care refers to the process of managing one’s health through self-care maintenance, symptom perception, and self-care management. It encompasses the behaviors involved in sustaining the stability of physiological health (self-care maintenance), monitoring and recognizing symptoms (symptom perception), and managing the onset of symptoms (self-care management).

**Core components of HF self-care**. The core components of HF self-care were derived from a practical guideline organized around key aspects of HF self-care (self-care maintenance, symptom perception, and self-care management) [36]. The recommendations focus primarily on the following areas: body weight and nutritional status, fluid and salt intake, exercise tolerance, smoking cessation and prevention of excessive drinking, immunization, medication adherence, psychological status, sleep, travel, leisure activity, and sexual activity, symptom monitoring, and appropriate responses to symptoms. Accordingly, the intervention content was designed by adopting these recommendations. Additionally, the content was adjusted in accordance with the recommendations provided by the Chinese guidelines for HF management [37, 38].

**Program theory.** The CSM was adopted as the guiding framework for the design of the intervention. As mentioned above, illness perceptions and self-efficacy are the crucial variables of this model. Therefore, this study targeted the effects of illness perceptions and self-efficacy on participants’ self-care behaviors and disease outcomes in the intervention design.

Illness perception includes both cognitive and emotional aspects. Cognitive perceptions are proposed to regulate objective health threats, while emotional perceptions are regarded as adjusters of negative emotions. There are eight dimensions of cognitive perceptions: identity, consequences, causes, timeline, personal control, treatment control, and illness coherence. Each dimension is independent but also interrelated. Emotional perceptions capture the negative emotional responses evoked by illness, and they can independently influence coping procedures and disease outcomes alongside cognitive perceptions [21]. Self-efficacy is posited to moderate the effects of cognitive and emotional perceptions on the enactment of coping procedures. Researchers suggest that when people’s representations of their illness evoke awareness such that they enact coping behaviors, they also form or activate representations of these coping behaviors, which include beliefs about the performance of particular behaviors [21].

**Evidence base.** We conducted a systematic review to evaluate the effectiveness of nurse-led self-care interventions for people with HF and identify potentially effective characteristics to inform intervention development [7, 8].

Our systematic review indicated that the core components of effective self-care interventions typically include assessment, self-care knowledge and skills, problem-solving, decision-making, goal-setting, action-planning, reminders, monitoring, feedback, and social support. These elements have been shown to promote behavior changes and improve patient outcomes. On the basis of subgroup analysis, we determined that the optimal duration for self-care interventions is usually one to three months. We chose a 6-week duration with reference to Jiang et al. [39], as previous research has shown this timeframe to yield the greatest improvements in health outcomes for HF patients. Our review also indicated that nurse-led self-care interventions are often delivered to individual participants via face-to-face approach in hospitals and telephone calls after discharge. Thus, we provided individual participants with face-to-face discharge education during their hospital stay and followed up with phone calls after discharge. The review suggested that effective face-to-face interventions typically include one to five sessions, each lasting 15 to 90 min, occurring weekly or biweekly. Follow-up sessions were recommended to be between two to eight, lasting 10 to 45 min, and scheduled twice a week to monthly. Owing to the short hospital stays and patients’ unstable conditions early in their admission, we could only provide one predischarge session, which was set to last one hour to cover the necessary content. To offer ongoing support and monitor adherence to self-care without overwhelming participants, we conducted one 20-minute reinforcement telephone follow-up session in the first week after discharge, followed by two more 20-minute sessions every two weeks.

**Conceptualization of the study intervention.** The potentially effective strategies identified in our previous systematic review were adopted to address illness perceptions and self-efficacy (Supplementary Table 1). The theoretical framework underpinning the proposed program is described in Supplementary Fig. 1. We anticipated that improvements in illness perceptions and self-efficacy would subsequently enhance the participants’ self-care behaviors and improve their prognosis, such as health-related quality of life, depression, anxiety, symptom burden, sleep quality, healthcare service utilization, and mortality.

**Description of the study intervention.** The 6-week nurse-led CSM of the self-regulation-based HF self-care program consisted of one 60-min face-to-face individual education session in the hospital (week 1) followed by one 20-min weekly (week 2) and two 20-min biweekly (week 4 and week 6) reinforcement telephone follow-up sessions after discharge (Supplementary Table 2). All the sessions were uniformly delivered by the principal investigator to ensure consistency.

The participants received educational handbooks and self-care logbooks (Supplementary Fig. 2). The education handbook contains three sections, namely HF disease management information, HF self-care knowledge and skills training, and behavior change techniques. The HF disease management information section gave information on the definition, causes, clinical manifestations, physical and psychological impacts, course of HF, and on treatments for HF. The HF self-care knowledge and skills training section focused on key HF self-care topics, including medication management, fluid management, nutrition management, exercise and rehabilitation management, smoking cessation, alcohol restriction and abstinence, emotional management, sleep management, leisure and entertainment, immunization and prevention of infections, and symptom monitoring, recognition, and management. The behavior change techniques section presents details on problem-solving, decision-making, goal-setting, and action-planning. The self-care logbook included structured forms (a goal-setting and action-planning record sheet, a problem-solving skills practice sheet, a medication list, a health record sheet, an exercise record sheet, and a fluid intake record sheet) to help participants adhere to self-care. A panel of experts in the field of cardiovascular care and a group of laypeople reviewed the education handbook and self-care logbook. The content validity of the education handbook and self-care logbook was evaluated using the content validity index (CVI) [40]. The item-level CVI, scale level-CVI/universal agreement, and scale level-CVI/Average all scored one in our study, indicating strong content validity. Participants also reported that the intervention contents were clear and understandable.

### Pilot testing

**Baseline data of the participants.** The mean age of the participants was 60.23 years (SD = 13.44), with the majority being male (69.2%). Only four subjects (15.4%) had tertiary education or higher, and more than half of the patients (69.2%) were unemployed. Almost all participants were married (96.2%), most dwelt in urban areas (92.3%), and all resided with others. Tables 1 and 2 present the characteristics of the participants and the study outcomes from the pilot study. There were no significant between-group differences in participants’ characteristics or study outcomes.

**Table 1:** Sociodemographic and clinical characteristics of participants in the pilot study (*N* = 26)

Characteristics	Total (*n* = 26) Mean ± SD/*n* (%)/Median (IQR)	Intervention (*n* = 13) Mean ± SD/*n* (%)/Median (IQR)	Control (*n* = 13) Mean ± SD/*n* (%)/Median (IQR)
**Sociodemographic characteristics**			
Age (years)	60.23 ± 13.44	60.31 ± 14.41	60.15 ± 12.99
Gender			
Male	18 (69.2)	10 (76.9)	8 (61.5)
Female	8 (30.8)	3 (23.1)	5 (38.5)
Marital status			
Married	25 (96.2)	12 (92.3)	13 (100)
Single/Divorced/Separated/Widowed	1 (3.8)	1 (7.7)	0 (0.0)
Educational level			
Primary or less	12 (46.2)	5 (38.4)	7 (53.8)
Secondary	10 (38.4)	6 (46.2)	4 (30.8)
Tertiary or above	4 (15.4)	2 (15.4)	2 (14.4)
Employment status			
Employed	8 (30.8)	5 (38.5)	3 (23.1)
Unemployed	18 (69.2)	8 (61.5)	10 (76.9)
Place of residence			
Urban	24 (92.3)	12 (92.3)	12 (92.3)
Rural	2 (7.7)	1 (7.7)	1 (7.7)
Living arrangement			
Living alone	0 (0.0)	0 (0.0)	0 (0.0)
Living with others	26 (100.0)	13 (100.0)	13 (100.0)
Smoking history			
No	15 (57.7)	7 (53.8)	8 (61.5)
Ex-smoker	2 (7.7)	1 (7.7)	1 (7.7)
Smoking	9 (34.6)	5 (38.5)	4 (30.8)
Drinking history			
No	18 (69.2)	9 (69.2)	9 (69.2)
Ex-drinker	2 (7.7)	1 (7.7)	1 (7.7)
Drinking	6 (23.1)	3 (23.1)	3 (23.1)
**Clinical Characteristics**			
Number of hospitalization (time)	3.38 ± 1.75	3.23 ± 1.69	3.54 ± 1.85
Time since diagnosis (month)	3.50 (1.00-27.75)	10.00 (1.50–31.50)	2.00 (1.00–37.00)
LVEF (%)	47.19 ± 15.00	47.38 ± 16.18	47.00 ± 14.39
NYHA functional classification			
Class I to II	11 (42.3%)	6 (46.2%)	5 (38.5%)
Class III to IV	15 (57.7%)	7 (53.8%)	8 (61.5%)
CCI (Possible score range: 0 ~ 37)	3.73 ± 1.66	4.08 ± 1.66	3.38 ± 1.66
BMI (kg/m^2^)	24.85 ± 4.10	24.19 ± 2.73	25.52 ± 5.15
SBP (mmHg)	118.27 ± 20.36	114 ± 19.99	122 ± 20.61
DBP (mmHg)	75.88 ± 14.48	75.92 ± 13.99	75.85 ± 15.53
HR (beat per minute)	78.69 ± 18.47	79.85 ± 16.20	77.54 ± 21.10
NT-ProBNP (pg/ml)	4892.35 ± 7521.40	4806.69 ± 8579.69	4978.00 ± 6650.58
TC (mmol/L)	4.32 ± 2.02	4.17 ± 0.95	4.46 ± 2.74
TG (mmol/L)	1.08 ± 0.49	1.14 ± 0.57	1.02 ± 0.411
HDL (mmol/L)	1.13 ± 0.39	1.05 ± 0.14	1.21 ± 0.53
LDL (mmol/L)	2.67 ± 1.42	2.62 ± 0.72	2.72 ± 1.93
HbA1c (%)	6.82 ± 1.56	6.95 ± 1.74	6.70 ± 1.42
Frailty* (Possible score range: 0 ~ 15)	5.38 ± 1.60	5.69 ± 1.70	5.08 ± 1.50

Note: LVEF, Left ventricular ejection fraction; NYHA, New York Heart Association; CCI, Charlson Comorbidity Index; BMI, Body mass index; SBP, Systolic blood pressure; DBP, Diastolic blood pressure; HR, Heart rate; TC, Total cholesterol; TG, Triglyceride; HDL, High density lipoprotein cholesterol; LDL, Low density lipoprotein cholesterol; HbA1c, Glycated hemoglobin. * Lower scores imply better outcome

**Table 2:** Baseline outcome variables of participants in the pilot study (*N* = 26)

Outcome variables (measures)	Total (*n* = 26) Mean ± SD	Intervention (*n* = 13) Mean ± SD	Control (*n* = 13) Mean ± SD
Illness perceptions (B-IPQ)* (Possible score range: 0 ~ 80)	46.46 ± 6.65	48.15 ± 6.69	44.77 ± 6.42
Self-care self-efficacy (SCSES) (Possible score range: 0 ~ 100)	37.31 ± 12.43	36.73 ± 11.24	37.88 ± 13.95
Self-care behaviors (SCHFI)			
Self-care maintenance (Possible score range: 0 ~ 100)	24.62 ± 11.89	24.81 ± 9.65	24.42 ± 14.18
Symptom perception (Possible score range: 0 ~ 100)	20.90 ± 11.39	23.41 ± 12.04	18.39 ± 10.56
Self-care management (Possible score range: 0 ~ 100)	38.35 ± 7.57	38.23 ± 5.87	38.46 ± 9.21
Health-related quality of life (MLHFQ) * (Possible score range: 0 ~ 105)	44.58 ± 11.89	47.77 ± 9.66	41.38 ± 13.39
Depression (PHQ-9) * (Possible score range: 0 ~ 27)	5.65 ± 2.50	6.00 ± 2.45	5.31 ± 2.59
Anxiety (GAD-7)* (Possible score range: 0 ~ 21)	5.19 ± 2.59	5.77 ± 3.03	4.62 ± 2.02
Symptom burden* (MSAS-HF) (Possible score range: 0 ~ 4)	0.65 ± 0.32	0.66 ± 0.35	0.64 ± 0.30
Sleep quality (PSQI)* (Possible score range: 0 ~ 21)	11.46 ± 3.88	12.54 ± 3.69	10.38 ± 3.91

Note: B-IPQ, Brief Illness Perception Questionnaire; SCHFI, Self-Care of Heart Failure Index; SCSES, Self-Care Self-Efficacy Scale; MLHFQ, Minnesota Living with Heart Failure Questionnaire; PHQ-9, 9-item Patient Health Questionnaire; GAD-7, 7-item Generalized Anxiety Disorder; MSAS-HF, Memorial Symptom Assessment Scale-Heart Failure; PSQI, Pittsburgh Sleep Quality Index. * Lower scores imply better outcome

**Feasibility of subject recruitment and retention.** A total of 34 potential participants were identified through medical records. Seven patients were excluded based on the eligibility criteria, and one declined to participate. Finally, 26 participants were enrolled and signed the informed consent form. The eligibility rate was 79.4% (27/34) and the recruitment rate was 96.3% (26/27). The reasons for exclusion included chronic kidney disease stage 4 (*n* = 3), inability to communicate (*n* = 2), recent participation in other research (*n* = 1), and mobility issues (*n* = 1).

**Fig. 1:**
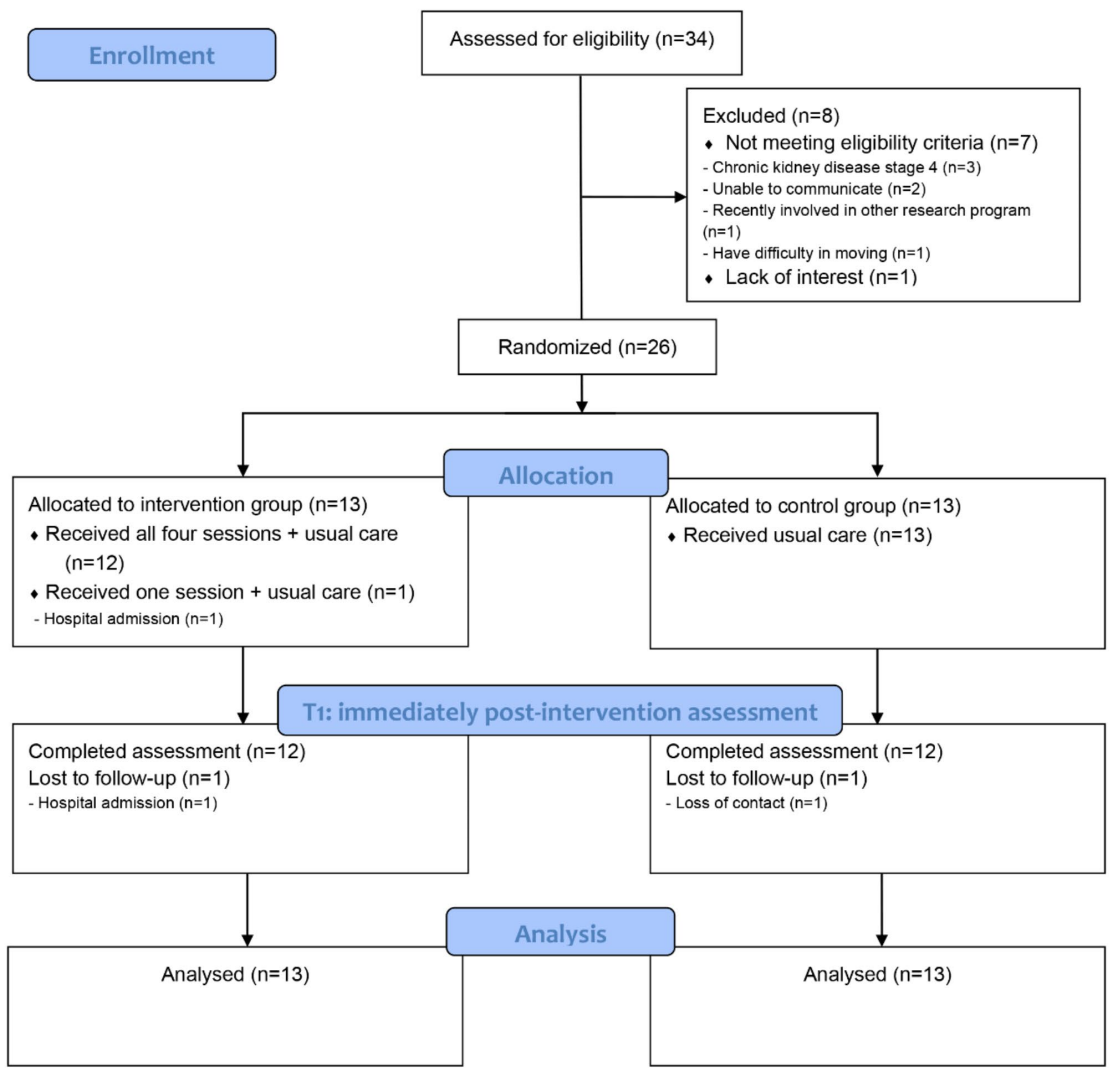
CONSORT flow chart of the pilot study

After collecting baseline data, all 26 enrolled participants were randomly assigned to either the intervention group (*n* = 13) or the control group (*n* = 13). One participant in the intervention group withdrew due to an acute exacerbation of HF that required hospitalization, and one subject in the control group was lost to follow-up after not answering phone calls. Ultimately, 24 participants completed the immediate post-intervention assessment, resulting in a retention rate of 92.3% (24/26) and an attrition rate of 7.7% (2/26) for this pilot study (Fig. 1).

**Feasibility and acceptability of the intervention**. All the eligible participants received the discharge education session while hospitalized, and all but one participant completed all the reinforcement telephone follow-up sessions. The participant who did not complete the final telephone session had been readmitted to the hospital and was therefore unable to do so. Therefore, 92.3% (12/13) of the participants completed the entire intervention. The median times for discharge education session was 60 min (IQR: 47.5 to 60 min), while the median times for the three reinforcement telephone follow-up sessions were 20 min (IQR: 15–20), 15 (IQR: 15–20) minutes, and 15 (IQR: 15–20) minutes.

Twelve participants completed a questionnaire to assess the intervention’s acceptability. All but two participants agreed or strongly agreed with all the items listed in the questionnaire, indicating an acceptance rate of 83.3%. None rated any items as strongly disagreed or disagreed (Supplementary Table 3). In addition, four participants provided suggestions on this program. One suggested making the recommended foods for individuals with heart failure more specific, so we added more examples to the educational handbook. The other three felt it was unnecessary to record their performance in the self-care logbook, even after completing the tasks.

**Feasibility of data collection.** Overall, the data collection process via face-to-face and telephone interviews went smoothly. The median times used to collect T0 and T1 data was 40 (IQR: 30–40) and 20 (IQR: 15–25) minutes, respectively.

**Potential effects.** Compared with the controls, the participants in the intervention group had statistically significant improvements in illness perceptions (β=-11.837, 95% CI: -16.747, -6.928, *P* < 0.001), self-care self-efficacy (β = 9.838, 95% CI: 6.088, 13.588, *P* < 0.001), self-care maintenance (β = 10.969, 95% CI: 1.238, 20.699, *P* = 0.027), symptom perception (β = 17.422, 95% CI: 10.265, 24.579, *P* < 0.001), and self-care management (β = 17.231, 95% CI: 10.279, 24.184, *P* < 0.001). However, there were no significant changes in health-related quality of life, depression, anxiety, symptom burden, or sleep quality (Table 3). The Mann–Whitney U test showed no significant difference in the number of HF-related hospital readmissions between the two groups (*P* = 0.317). No deaths or other healthcare service utilization outcomes were reported.

**Table 3:** Generalized estimating equations analysis for comparison of the outcome variables between the intervention and control groups

Outcome	Time point	Intervention group	Control group	Group effect		Time effect		Group×time effect	
				β (95% CI)	P	β (95% CI)	P	β (95% CI)	P
Illness perceptions	T0	48.15 ± 6.69	44.77 ± 6.42	3.385 (-1.095, 7.865)	0.139				
	T1	23.42 ± 5.52	32.00 ± 4.20			-13.050 (-16.521, -9.578)	< 0.001	-11.837 (-16.747, -6.928)	< 0.001
Self-care self-efficacy	T0	36.73 ± 11.24	37.88 ± 13.95	-0.462 (-3.758, 2.835)	0.784				
	T1	40.17 ± 3.51	30.42 ± 2.91			5.517 (2.866, 8.168)	< 0.001	9.838 (6.088, 13.588)	< 0.001
Self-care maintenance	T0	24.81 ± 9.65	24.42 ± 14.18	0.385 (-7.150, 7.919)	0.920				
	T1	50.63 ± 8.20	38.75 ± 3.61			14.852 (7.971, 21.732)	< 0.001	10.969 (1.238, 20.699)	0.027
Symptom perception	T0	23.41 ± 12.04	18.39 ± 10.56	5.017 (-2.481, 12.514)	0.190				
	T1	60.73 ± 7.15	37.12 ± 8.10			19.712 (14.651, 24.773)	< 0.001	17.422 (10.265, 24.579)	< 0.001
Self-care management	T0	38.23 ± 5.87	38.46 ± 9.21	-0.233 (-6.199, 5.732)	0.939				
	T1	76.52 ± 7.77	59.34 ± 7.80			21.451 (16.534, 26.367)	< 0.001	17.231 (10.279, 24.184)	< 0.001
Health-related quality of life	T0	47.77 ± 9.66	41.38 ± 13.39	6.385 (-0.659, 13.428)	0.076				
	T1	13.25 ± 6.28	12.75 ± 3.74			-28.316 (-35.026, -21.607)	< 0.001	-6.100 (-15.589, 3.388)	0.208
Depression	T0	6.00 ± 2.45	5.31 ± 2.59	0.692 (-0.877, 2.262)	0.387				
	T1	1.25 ± 1.14	1.33 ± 1.50			-3.988 (-5.082, -2.895)	< 0.001	-0.671 (-2.218, 0.876)	0.395
Anxiety	T0	5.77 ± 3.03	4.62 ± 2.02	1.154 (-0.436, 2.744)	0.155				
	T1	0.83 ± 1.19	0.83 ± 1.40			-3.853 (-4.980, -2.726)	< 0.001	-1.073 (-2.667, 0.522)	0.187
Symptom burden	T0	0.66 ± 0.35	0.64 ± 0.30	0.022 (-0.164, 0.209)	0.814				
	T1	0.11 ± 0.06	0.11 ± 0.10			-0.522 (-0.685, -0.359)	< 0.001	-0.023 (-0.253, 0.207)	0.845
Sleep quality	T0	12.54 ± 3.69	10.38 ± 3.91	2.154 (-0.488, 4.796)	0.110				
	T1	7.50 ± 3.55	6.0 ± 2.30			-4.408 (-5.817, -3.000)	< 0.001	-0.541 (-2.533, 1.451)	0.595

Note: T0, baseline; T1: immediately post-intervention

## Discussion

In alignment with the MRC framework, this study details the development of a nurse-led CSM of Self-Regulation-based HF self-care program and tests its feasibility, acceptability, and potential effects. The findings indicated that the program was theory-driven, evidence-based, and tailored to meet participants’ needs. The pilot study indicated that the intervention was feasible, acceptable, and potentially beneficial for people with HF.

We adopted the CSM of Self-Regulation to guide the design of this intervention because it effectively improved health behaviors and outcomes across various conditions. Underpinned by this model, we conducted a systematic review to identify the potentially effective characteristics of nurse-led self-care interventions, including intervention content, dosage, format, and delivery modes. These strategies were clearly detailed in our study, aiding in understanding the intervention’s effects and facilitating replication in other settings. Moreover, the detailed content of our intervention was developed in accordance with the current guidelines and validated by cardiovascular experts and participants. Most importantly, the delivery of the intervention content was tailored to respond to the needs of participants.

The eligibility rate for this pilot study was 79.4%, which is relatively high and indicates that the eligibility criteria were appropriate. The study achieved a high recruitment rate of 96.3% and a retention rate of 92.3%, suggesting strong interest among participants. The findings revealed that all but one participant completed the full intervention sessions, and the majority of participants expressed satisfaction with the program, collectively indicating that the interventions were feasible and acceptable to the target population. Notably, two participants were neutral about the intervention’s effects on sleep quality due to chronic sleep issues, and one participant did not express a clear opinion of the benefits for HF symptoms, likely because of poorer cardiac function compared to others. During the intervention, we found that relying solely on self-care logbook records did not guarantee adherence to the prescribed activities. Therefore, it is essential to emphasize the importance of adherence and to engage participants in discussions about their HF self-care practices, rather than just reviewing logbook entries.

This study provided preliminary evidence of the favorable effects of our program. It is not surprising to observe improved illness perceptions, self-care self-efficacy, and self-care behaviors among participants in the intervention group, as we employed appropriate strategies to address the key factors influencing self-care behaviors based on the CSM of Self-Regulation. Our program offers HF self-care knowledge and skills, along with behavior change techniques such as goal-setting, action-planning, decision-making, problem-solving, and continuous support. These elements help participants adjust their beliefs about HF and enhance their understanding of and control over their condition. The tailored self-care recommendations enabled participants to acquire specific self-care knowledge and skills, boosting their confidence in self-care. They were guided to create action plans for fulfilling self-care tasks and use problem-solving skills for daily challenges, making them more capable of handling their condition. Improvements in illness perceptions and self-care self-efficacy contribute to better self-care behaviors. The lack of significant effects on other study outcomes may be attributed to an insufficient sample size to detect the intervention’s impact. In this pilot study, the primary objective was to evaluate the feasibility and acceptability of the intervention program.

Despite the positive outcomes from the pilot study, we acknowledge that the results may be limited by our exclusive use of a quantitative approach. The incorporation of qualitative interviews to explore participants’ experiences with the program could increase the richness and depth of the study findings. Therefore, a mixed-method RCT is warranted in further studies to gain an in-depth understanding of our program. In addition, the representativeness of our sample may have been affected by an unbalanced distribution of gender and age. Since we have developed a robust nurse-led HF self-care program that has shown feasibility, acceptability, and potential benefits, the next step is to conduct a full-scale RCT to rigorously examine its effectiveness.

## Conclusion

This study piloted a nurse-led, theory-driven, evidence-based, and need-oriented self-care intervention for individuals living with HF. The findings from the pilot study supported the feasibility, acceptability, and potential effects of the intervention, underscoring the necessity for a full-scale RCT with a process evaluation to further examine its effectiveness.

## Electronic supplementary material

Below is the link to the electronic supplementary material.

Supplementary Material 1

Supplementary Material 2

## Data Availability

Data cannot be shared openly but are available on request from authors.

## Abbreviations

CSM: Common-Sense Model
CVI: Content validity index
GEE: Generalized estimating equation
HF: Heart failure
IQR: Interquartile range
RCT: Randomized controlled trial
MRC: Medical Research Council
NYHA: New York Heart Association
