# Supplementary material for: Development and pilot testing of a nurse-led common-sense model of self-regulation-based heart failure self-care program
Source: BMC Nurs. 2025 Jan 23;24:85. doi: 10.1186/s12912-025-02722-9 (PMC11758749; doi:10.1186/s12912-025-02722-9)
Supplement: Supplementary file 1 — Supplementary Material 1 [file 12912_2025_2722_MOESM1_ESM.docx]

Table 1 Strategies adopted to improve participants’ illness perceptions and self-efficacy

| **Variables** | **Components** | **Strategies** | **Variables** |
| --- | --- | --- | --- |
| Illness perceptions | Causes | - Introduce the causes of HF | Self-efficacy |
|  | Consequences | - Introduce the physical and psychosocial impacts of HF |  |
|  | Identity | - Introduce the definition and symptoms and signs of HF |  |
|  | Timeline | - Introduce the prognosis of HF |  |
|  | Personal control | - Introduce the relationship between self-care and HF recovery  - Provide self-care knowledge and skill training  - Assist in using problem-solving skills to make decisions in the self-care process  - Assist in establishing self-care goals and action plans  - Provide positive feedback on personal actions  - Assist in solving the barriers and problems in the self-care process |  |
|  | Treatment control | - Introduce the relationship between HF treatments and disease control |  |
|  | Illness coherence | - Assist in understanding HF |  |
|  | Emotional perception | - Provide information to reduce the negative emotion caused by HF |  |

Table 2 Outline of the nurse-led CSM of self-regulation-based HF self-care program

| **Week** | **Themes** | **Goals** | **Contents** |
| --- | --- | --- | --- |
| 1 | Discharge education on HF self-care | (1) To assist participants in developing accurate perceptions of HF and confidence in self-care  (2) To emphasize the significance of self-care while equipping participants with essential self-care knowledge and skills  (3) To facilitate self-care among participants | - Implement individual assessments of the participants’ illness perceptions - Identify inaccurate perceptions of HF and provide information via education handbook to establish accurate illness perceptions - Emphasize the significance of self-care and perform individual assessments of the participants’ self-care self-efficacy and self-care behaviors - Identify problems with self-care and use the education handbook to provide the participants with self-care knowledge and skills training to enhance self-care self-efficacy and self-care behaviors - Teach the participants how to use problem-solving skill to make decisions on managing individual self-care problems using education handbook and self-care logbook - Guide the participants how to establish self-care goals and action plans using education handbook and self-care logbook |
| 2, 4, and 6 | Reinforcement telephone follow-up session | (1) To assess and monitor the participants’ self-care performance  (2) To reinforce the significance of self-care practices  (3) To encourage and support participants in their self-care | - Review the participants’ goal achievement - Provide positive feedback on the participants’ efforts to adhere to self-care - Point out any the discrepancies between current behaviors and goals - Discuss barriers to and problems in implementing self-care - Encourage the participants to continue to use problem-solving skills to make decisions in managing individual self-care problems - Encourage the participants to continue following action plans and working towards goals or to modify these, as appropriate - Remind the participants to use the education handbook and self-care logbook |

Table 3 Participants’ perceptions of the program

| **Your views on the program** | **Neither agree nor disagree** | **Strongly agree or agree** |
| --- | --- | --- |
| 1. Overall, I am pleased with the program. | 0% | 100% |
| 2. The contents of the program are comprehensive and easy to understand. | 0% | 100% |
| 3. The number, duration, frequency, and delivery mode of the sessions are suitable. | 0% | 100% |
| 4. The intervention booklet and videos are well-designed and useful. | 0% | 100% |
| 5. I think that the program enhanced my perception of heart failure and self-care. | 0% | 100% |
| 6. I think that the program provided me with the knowledge and skills to manage my condition. | 0% | 100% |
| 7. I think that the program boosted my confidence to look after myself. | 0% | 100% |
| 8. I think that the program improved my quality of life. | 0% | 100% |
| 9. I think that the program alleviated my heart failure symptoms. | 8.3% | 91.7% |
| 10. I think that the program made me feel better. | 0% | 100% |
| 11. I think that the program improved my sleep quality. | 26.7% | 83.3% |
| 12. I would like to recommend the program to other patients. | 0% | 100% |

**
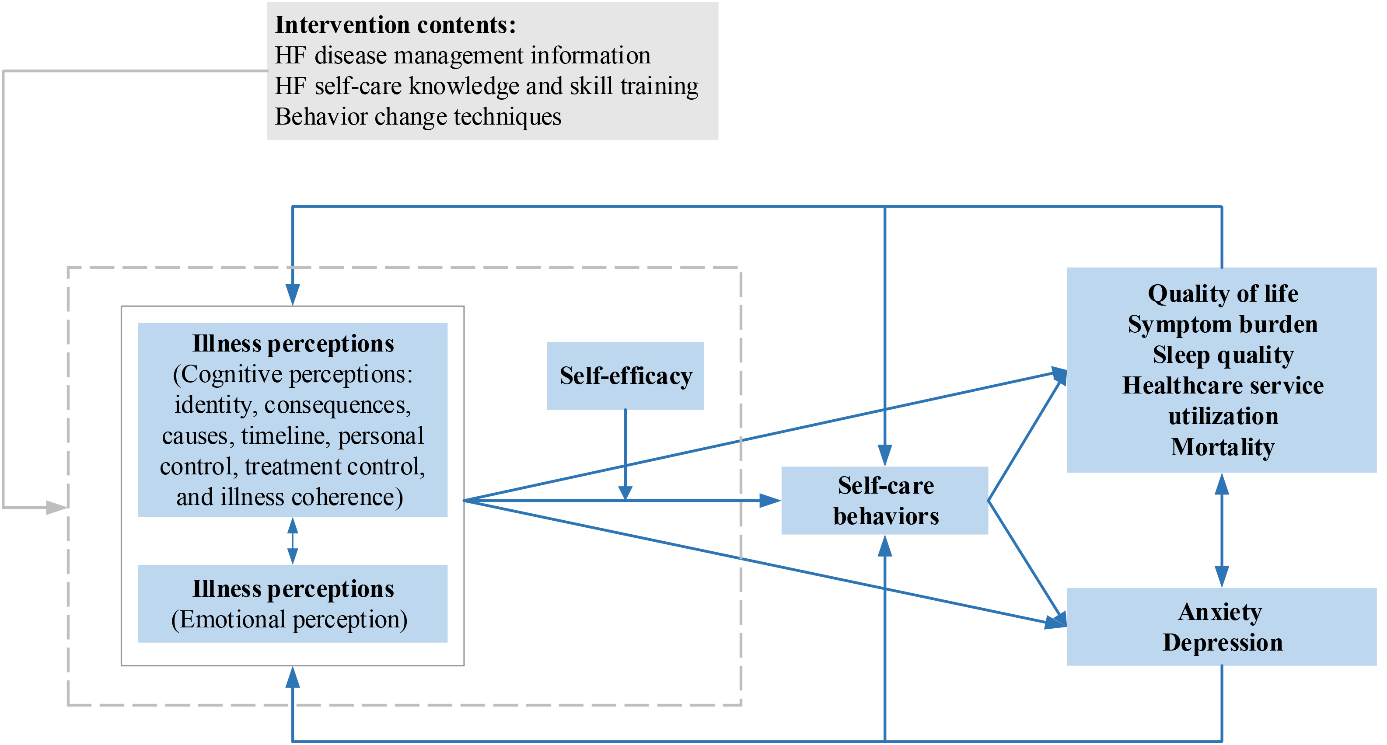
**

Figure 1 Theoretical framework underpinning the proposed program

**
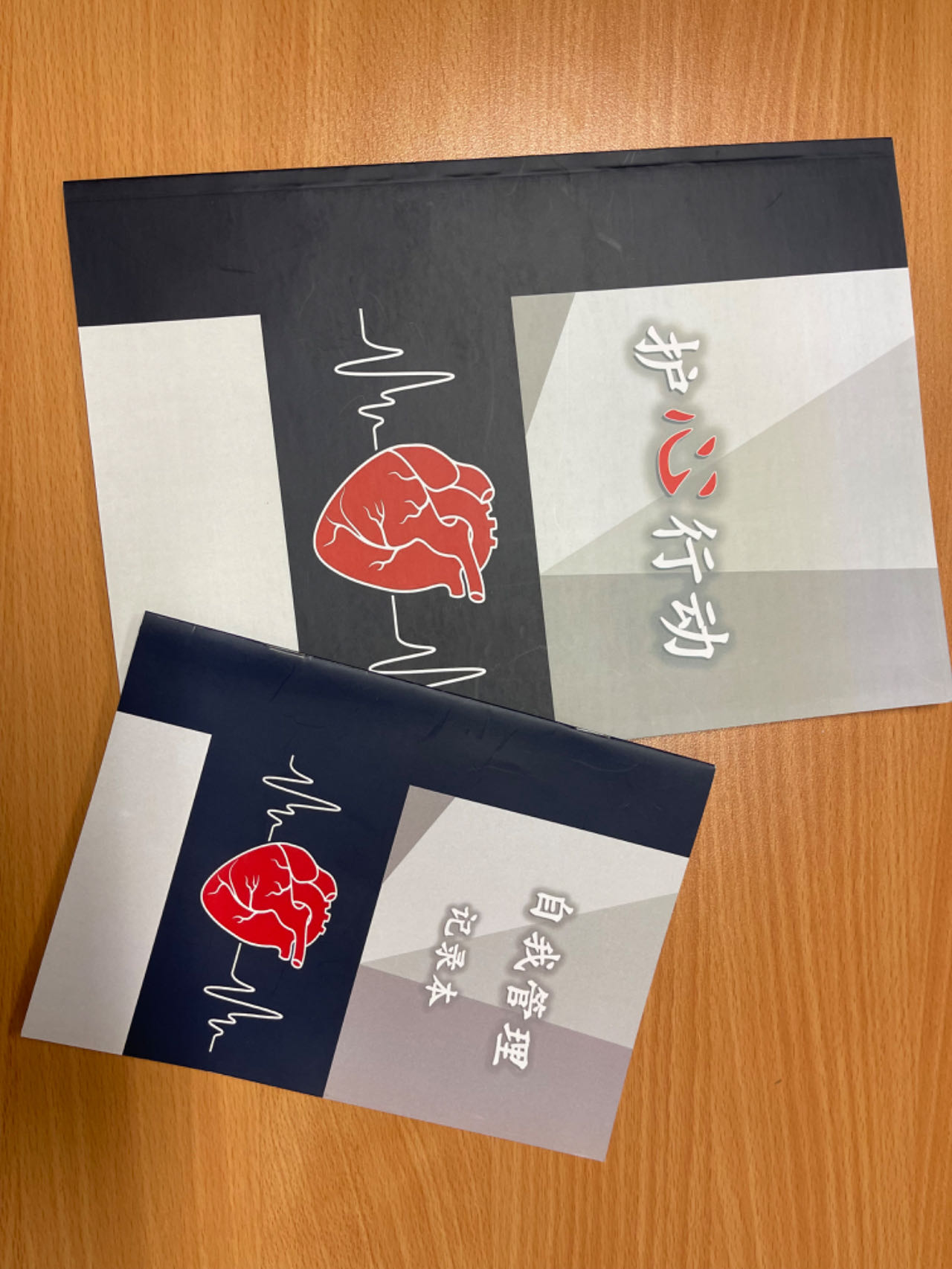
**

Figure 2 Education handbook (left) and self-care logbook (right)
